# Heme oxygenase-1 protects against Alzheimer's amyloid-*β*_1-42_-induced toxicity *via* carbon monoxide production

**DOI:** 10.1038/cddis.2014.529

**Published:** 2014-12-11

**Authors:** N Hettiarachchi, M Dallas, M Al-Owais, H Griffiths, N Hooper, J Scragg, J Boyle, C Peers

**Affiliations:** 1 Division of Cardiovascular and Diabetes Research, LICAMM Faculty of Medicine and Health, University of Leeds, Leeds, LS2 9JT, UK; 2 School of Molecular and Cellular Biology, Faculty of Biological Sciences, University of Leeds, Leeds, LS2 9JT, UK

## Abstract

Heme oxygenase-1 (HO-1), an inducible enzyme up-regulated in Alzheimer's disease, catabolises heme to biliverdin, Fe^2+^ and carbon monoxide (CO). CO can protect neurones from oxidative stress-induced apoptosis by inhibiting Kv2.1 channels, which mediates cellular K^+^ efflux as an early step in the apoptotic cascade. Since apoptosis contributes to the neuronal loss associated with amyloid *β* peptide (A*β*) toxicity in AD, we investigated the protective effects of HO-1 and CO against A*β*_1-42_ toxicity in SH-SY5Y cells, employing cells stably transfected with empty vector or expressing the cellular prion protein, PrP^c^, and rat primary hippocampal neurons. A*β*_1-42_ (containing protofibrils) caused a concentration-dependent decrease in cell viability, attributable at least in part to induction of apoptosis, with the PrP^c^-expressing cells showing greater susceptibility to A*β*_1-42_ toxicity. Pharmacological induction or genetic over-expression of HO-1 significantly ameliorated the effects of A*β*_1-42_. The CO-donor CORM-2 protected cells against A*β*_1-42_ toxicity in a concentration-dependent manner. Electrophysiological studies revealed no differences in the outward current pre- and post-A*β*_1-42_ treatment suggesting that K^+^ channel activity is unaffected in these cells. Instead, A*β* toxicity was reduced by the L-type Ca^2+^ channel blocker nifedipine, and by the CaMKKII inhibitor, STO-609. A*β* also activated the downstream kinase, AMP-dependent protein kinase (AMPK). CO prevented this activation of AMPK. Our findings indicate that HO-1 protects against A*β* toxicity *via* production of CO. Protection does not arise from inhibition of apoptosis-associated K^+^ efflux, but rather by inhibition of AMPK activation, which has been recently implicated in the toxic effects of A*β*. These data provide a novel, beneficial effect of CO which adds to its growing potential as a therapeutic agent.

Amongst the earliest of events leading to neuronal loss in Alzheimer's disease (AD) is the loss of functional synapses,^1, 2, 3^ apparent long before deposition of amyloid *β* peptide (A*β*)-containing plaques.^4^ Although other parts of the neurone (e.g. the axon or soma) appear intact, their health at this early stage of disease progression is not clear. However, neurones ultimately die in AD and there is clear evidence that numerous events indicative of apoptosis occur even at early stages of disease progression.^5, 6, 7, 8^ Thus, targeting of apoptotic mechanisms may be of therapeutic value in AD as well as in other neurodegenerative disorders. Furthermore, apoptosis is established as a mechanism of neuronal loss following other types of pathological stresses including ischemia associated with stroke,^9^ which can predispose individuals to the development of AD.^10, 11, 12^

Apoptosis is strongly influenced by intracellular K^+^ levels^13^ which regulate caspase activation, mitochondrial membrane potential and volume, osmolarity and cell volume.^13, 14^ K^+^ loss *via* K^+^ channels is a key early stage in apoptosis,^15, 16, 17, 18, 19^ and K^+^ channel inhibitors can protect against apoptosis triggered by numerous insults including oxidative stress.^20, 21^ Evidence suggests a particularly important role for the voltage-gated channel Kv2.1 in this process: expression of dominant negative Kv2.1 constructs (thus lacking functional Kv2.1 channels) protects against oxidant-induced apoptosis, and over-expression of Kv2.1 increases susceptibility to apoptosis.^22, 23^ Pro-apoptotic agents cause a rapid increase in the surface expression of Kv2.1 channels,^24^ but whether or not this occurs in AD remains to be determined. Alternative pathways recently reported to promote cell death include activation of the AMP-dependent protein kinase (AMP kinase) which can act either as a Tau kinase^25^ or to inhibit the mTOR pathway^26^ and thus contribute to neurodegeneration.

Heme oxygenases (HO) are enzymes widely distributed throughout the body. In the central nervous system, HO-2 is constitutively expressed in neurones and astrocytes, while HO-1 is inducible in both cell types.^27, 28, 29, 30^ Both HO-1 and HO-2 break down heme to liberate biliverdin, ferrous iron (Fe^2+^) and carbon monoxide (CO). This catalysis is of biological significance since it is crucial to iron and bile metabolism, and also generates a highly effective antioxidant in bilirubin (from biliverdin *via* bilirubin reductase). Numerous stimuli can induce HO-1 gene expression,^31^ including oxidative stress^32^ and A*β* peptides.^33^ Importantly, HO-1 is strikingly up-regulated in AD patients, a finding considered indicative of oxidative stress.^27, 34, 35^ Induction of HO-1 is clearly a neuroprotective response (although in some cases can exert detrimental effects^27^). However, there is growing evidence that CO can be neuroprotective, for example against the damage of focal ischemia.^36^ Our recent studies have demonstrated that CO provides protection against oxidant-induced apoptosis by selectively inhibiting Kv2.1.^23, 37^ In the present study, we have investigated whether HO-1, or its product CO, can provide protection against A*β*-induced toxicity in the human neuroblastoma, SH-SY5Y, and in rat primary hippocampal neurones, and whether this involves regulation of K^+^ channels. We show that both HO-1 and CO protect cells against the toxicity of protofibrillar A*β*_1-42_ but that protection does not arise from inhibition of apoptosis-associated K^+^ efflux, but rather by inhibition of AMPK activation.

## Results

### A*β*_(1-42)_-induced cell death in SH-SY5Y cells

**Figure 1:**
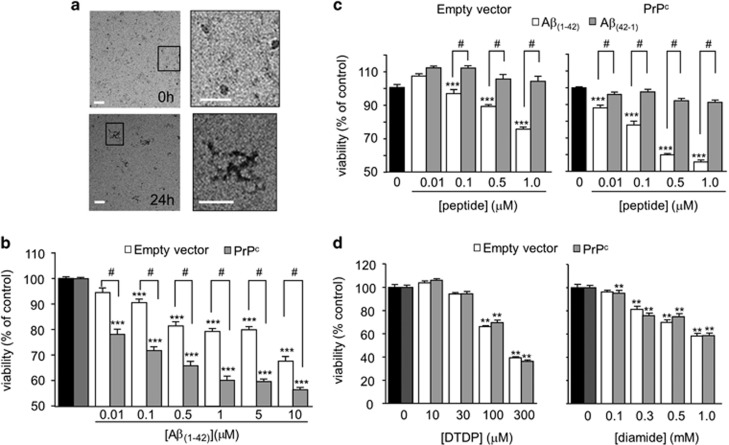
Aggregation of A*β*_1-42_ into protofibrils is toxic to SH-SY5Y cells. (**a**) Representative images seen by negative stain electron microscopy of globular aggregates seen in A*β* freshly dissolved in DMEM (0 h, upper images). Boxed area of left-hand image is magnified in right-hand image to show more detail of these structures. Lower images show globular aggregates plus protofibrillar structures in A*β* solutions stored at 37 ^o^C for 24 h. Boxed area of left-hand image is magnified in right-hand image to show more detail of these structures. Scale bar in all images 50 nm. (**b**) Effect of A*β*_1-42_ on cell viability in SH-SY5Y cells containing empty vector (white bars) and stably expressing PrP^c^ (gray bars) using the mitochondrial activity-based MTT assay. Bars represent the mean±S.E.M. data of cells from 10 repeats (each performed in duplicate) with cells from different passages. (**c**) The effect of different concentrations of A*β*_1-42_ (white bars) and the reverse sequence A*β*_42-1_ (gray bars) on cell viability in empty vector containing and PrP^c^-expressing cells. Bars show the mean±S.E.M. of four repeats. Statistical significance was determined with a one-way ANOVA followed by Bonferroni *post-hoc* test. ***P<0.001, ^#^*P*<0.01. (**d**) Effect of oxidants on cell viability in empty vector containing (white bars) and PrP^c^-expressing (gray bars) cells. Left, effect of different concentrations of DTDP (10 min exposure, left) on cell viability. Each bar represents the mean±S.E.M. of seven repeats. Right, cell viability following a 30 min exposure to various concentrations of diamide. Each bar shows the mean±S.E.M. of five repeats using cells from different passages. Statistical significance is denoted by **P<0.01, compared to the corresponding control cells

To investigate any potential role of HO-1 and CO in affording protection against toxicity induced by A*β*_(1-42)_ (hereafter referred to simply as A*β*), we first determined their effectiveness in SH-SY5Y cells either expressing the cellular prion protein (PrP^c^) or containing the empty vector, as PrP^c^ is a receptor for oligomeric^38, 39^ and protofibrillar^40^ forms of A*β*. Freshly dissolved A*β* which contained small globular structures (<10 nm) and A*β* monomers (Figure 1a, upper images) had no effect on cell viability (not shown). After 24 h incubation at 37 °C, in addition to the small globular assemblies and monomers, the A*β* had formed protofibrils (25–90 nm in length) as assessed by electron microscopy (Figure 1a, lower images). These structures closely resembled the nanotubes that have recently been shown to mediate PrP^c^-dependent and –independent synaptotoxicity.^40^ There was no evidence of any amyloid fibrils in our preparations. Using MTT assays to evaluate cell viability following exposure to A*β* for 24 h, we found that the protofibrillar A*β* caused a concentration-dependent loss of viability, and that cells over-expressing PrP^c^ were significantly more sensitive to A*β* toxicity than the cells lacking PrP^c^ (Figure 1b). Extending the incubation period to 48 h did not increase toxicity further (data not shown). Neither the PrPc-expressing nor the empty vector containing cells were significantly affected by the reverse sequence peptide (A*β*_(42-1)_) (Figure 1c). Consistent with the idea that PrP^c^ at least partly mediates aggregated A*β* toxicity,^38, 39, 40^ over-expression of PrP^c^ appeared to confer specific sensitivity to A*β* toxicity, since both the PrPc expressing and empty vector containing cells were similarly sensitive to the oxidizing agents dithiodipyridine (DTDP) and diamide (Figure 1d), both of which have previously been shown to induce apoptosis.^23, 37^

### A*β*-induced cell death is at least partly attributable to apoptosis

**Figure 2:**
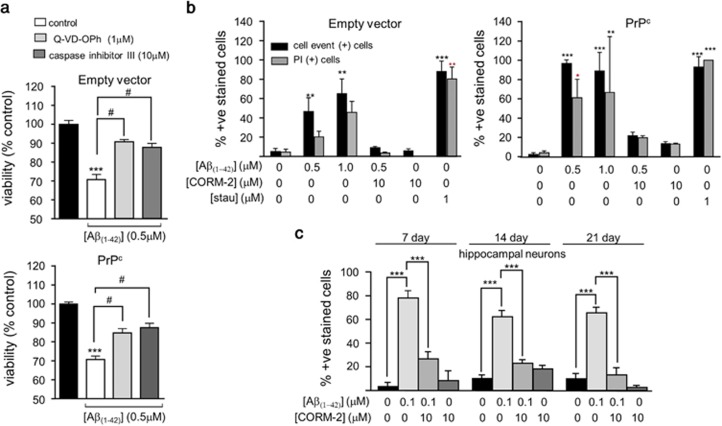
Amyloid toxicity is partly attributable to induction of apoptosis and reversed by CORM-2. (**a**) Effect on cell viability in empty vector containing (upper) and PrP^c^-expressing (lower) cells following 24 h treatment with 0.5 *μ*M A*β*_1-42_ alone or in the presence of either of two different caspase inhibitors; Q-VD-OPh (1 *μ*M) and caspase inhibitor III (10 *μ*M), as indicated. Black bars represent untreated cells. Bars indicate mean±S.E.M. of three repeats with cells from different passages for empty vector containing cells and four repeats for the PrP^c^-expressing cells. Statistical significance is denoted by ***P<0.001 when compared with the corresponding untreated control and ^#^*P*<0.001 when compared with the corresponding A*β*_1-42_ treated cells. (**b**) CellEvent positive cells indicate activated caspase 3. Black bars represent the percentage of CellEvent positive cells and gray bars represent the percentage of PI-positive cells compared with Hoescht positive cells in the area counted. Treatments were for 24 h with the concentrations of drugs indicated. As a positive control, cells were also treated with 1 *μ*M staurosporine for 10 min and stained with CellEvent and PI. Experiments were performed in triplicate and repeated with cells from three different passages. Statistical significance is denoted by **P<0.01 and ***P<0.001 when compared with the corresponding untreated cells. (**c**) Summary of the CellEvent staining experiments for 7, 14 and 21 day old hippocampal neurons, as indicated. Bars represent the percentage of CellEvent positive cells compared to the total number of Hoechst positive cells in the area counted. Cells were treated for 24 h with 100 nM A*β*_1-42_, 100 nM A*β*_1-42_+10 *μ*M CORM or 10 *μ*M CORM alone, as indicated. Experiments were performed in triplicate and repeated with cells from three different isolations and shown as mean±S.E.M. Statistical significance is denoted by ***P<0.001 compared with the untreated cells of that specific age group

To investigate whether A*β*-induced toxicity arose from induction of apoptosis, two separate approaches were taken. The reduction in cell viability caused by A*β* was significantly reduced by two distinct caspase inhibitors, the pan-caspase inhibitor (Q-VD-OPh; 1 *μ*M) or the irreversible caspase inhibitor III (Boc-D-FMK; 10 *μ*M) (Figure 2a), consistent with the idea that A*β* toxicity involves activation of apoptosis. In both PrPc-expressing cells and empty vector containing cells, A*β* caused an increase in the proportion of both CellEvent positive and PI-positive cells (i.e. cells showing increased caspase activity and loss of viability), approaching levels seen in cells treated with staurosporine, which induced positive caspase and PI staining in 80–100% of cells (Figure 2b). These data further support an important role for apoptosis induction in the A*β*−induced loss of cell viability. Using this same assay, we also observed that the CO-donor, CORM-2, significantly attenuated the number of CellEvent and PI-positive cells following exposure to 0.5 *μ*M A*β* (Figure 2b), suggesting a possible protective role for CO against A*β* toxicity. Importantly, we confirmed this potentially protective effect of CO in cultured rat primary hippocampal neurones (Figure 2c). Thus, employing cultures ranging from 7–21 days *in vitro*, we found that the same protofibrillar preparation of A*β* at a concentration of 100 nM caused marked increases in the number of apoptotic cells (indicated by CellEvent positive cells). Effects were found at all culture ages, and A*β* was more potent in inducing apoptosis in hippocampal neurones than in the SH-SY5Y cells (Figure 2c). In the presence of CORM-2 (10 *μ*M), the effects of A*β* on hippocampal neurones were largely reversed, and CORM-2 was without significant effect itself on apoptosis (Figure 2c). These findings indicate that the neuroprotective effects of CO against A*β* toxicity can be observed in different neuronal preparations.

### HO-1 induction protects against A*β* toxicity

**Figure 3:**
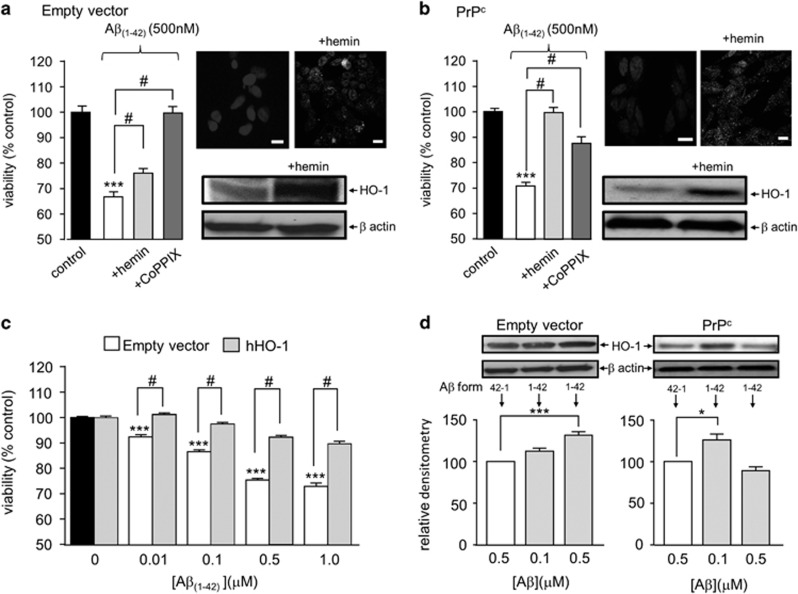
HO-1 protects against A*β* toxicity. (**a**) Cell viability measured in empty vector containing cells following 24h induction of HO-1 with either 30 *μ*M hemin or 3 *μ*M CoPPIX in the presence of 500 nM A*β*. Bars indicate mean±S.E.M. of four repeats with cells from different passages. Right (upper) immunofluorescent images of untreated empty vector containing cells (left) and cells treated with 30 *μ*M hemin (right) for 24 h and then probed using anti-HO-1 primary antibody and DAPI for nuclear staining. Lower; western blot detection of HO-1 following identical hemin induction. *β* actin blots are also shown to indicate even loading of protein. (**b**) As (**a**) except studies were conducted in PrP^c^-expressing cells. (**c**) Effect of different concentrations of A*β* on empty vector containing cells (black and clear bars) and cells stably over-expressing hHO-1. Bars represent mean±S.E.M. of six repeats (done in duplicate). (**d**) Detection of HO-1 induced by A*β* in both cell groups. Upper, example blots from cells exposed either to A*β*_42-1_ (used as a control) or A*β*_1-42_ at the two concentrations indicated in the bar graphs, below. These show mean densitometric analysis of induced expression in empty vector and PrP^c^-expressing cells. Bars represent mean±S.E.M. of four repeats (done in duplicate). Statistical significance is denoted by ***P<0.001 and *P<0.05 when compared with the corresponding untreated control and ^#^*P*<0.001 when compared to the corresponding A*β* treated cells

In order to investigate the ability of HO-1 to protect against A*β* toxicity, cells were exposed to two established inducers of this enzyme. Exposure to either 30 *μ*M hemin or 3 *μ*M CoPPIX for 24 h significantly attenuated the toxic effects of A*β* in both empty vector containing (Figure 3a) and PrPc-expressing SH-SY5Y cells (Figure 3b). HO-1 induction was verified by immunocytochemistry and western blotting in response to hemin (similar results obtained for CoPPIX; data not shown). Densitometric analysis indicated that hemin increased HO-1 expression to 188.0±5.0% of control levels (i.e. 1.88-fold; *P*<0.001, *n*=4 repeats) in empty vector containing cells, and by 239.5±28.8% (*P*<0.05, *n*=4 experiments) in PrPc-expressing cells. These data suggested that HO-1 affords protection against A*β* in addition to its known ability to protect against oxidant-induced apoptosis. To investigate this further, we over-expressed HO-1 in SH-SY5Y cells. As shown in Figure 3c, HO-1 over-expression also significantly diminished the toxic effects of A*β*. Since exposure to A*β* is clearly deleterious to cells, we also examined whether A*β* itself could induce expression of HO-1. As shown by western blotting in Figure 3d, 24 h exposure to A*β* did indeed increase expression of HO-1 in empty vector and PrPc-expressing cells, respectively. Interestingly, there was differential sensitivity to A*β* in the two cell groups: For empty vector cells, a graded increase in HO-1 induction was observed, but in PrPc-expressing SH-SY5Y cells significant induction was observed in response to 100 but not 500 nM A*β*. In both cell groups, induction was modest compared with the effects of hemin or CoPPIX.

**Figure 4:**
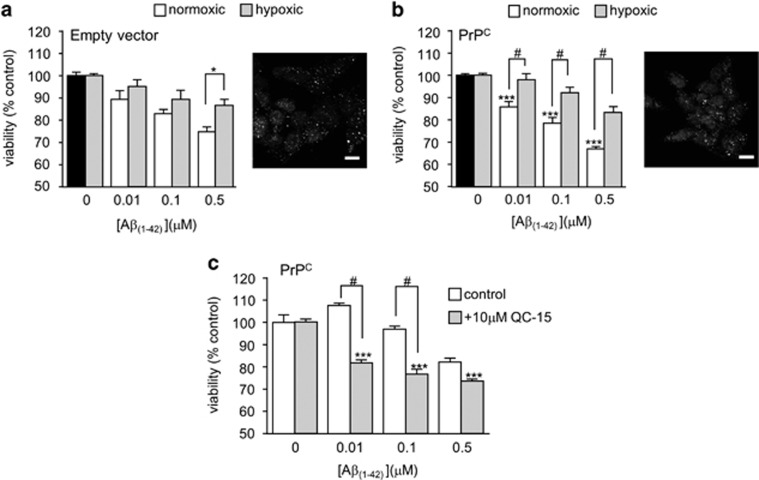
Protective effect of hypoxia against A*β* toxicity. (**a**) Effect of hypoxia (48 h, 0.5% O_2_) on A*β*-induced loss of cell viability in empty vector containing cells. Bars show mean±S.E.M. of four repeats. Right, immunofluorescent images using anti-HO-1 primary antibody showing upregulation of HO-1 following 48 h of hypoxia. (**b)** As (**a**) except studies were conducted in PrP^c^-expressing cells. (**c**) Effects of A*β* on cell viability in hypoxic PrP^c^-expressing cells in the absence (open bars) and presence (shaded bars) of the HO-1 inhibitor, QC-15. Bars represent the mean±S.E.M. of four repeats. Statistical significance is denoted by ***P<0.001, ^#^*P*<0.01 when compared with corresponding control and corresponding A*β*_1-42_ treated cells, respectively

Hypoxia has long been known to induce HO-1 expression,^41, 42^ so we investigated the sensitivity to A*β* of cells which had been maintained in a hypoxic environment (0.5% O_2,_ 24 h) prior to (and during) exposure to A*β*. Hypoxia did indeed induce HO-1 expression, as determined immunocytochemically, and hypoxic cells (both PrPc-expressing and empty vector containing cells) were significantly more resistant to the toxic actions of A*β* (Figures 4a and b). This was particularly prominent in the PrPc-expressing cells. We therefore used these cells exclusively to examine the ability of the HO-1 inhibitor QC-15^43^ to modulate the responses of hypoxia-exposed cells to A*β*. As shown in Figure 4c, QC-15 reversed the protective effects of hypoxia against A*β* toxicity, suggesting strongly that the protective effects of hypoxia were specifically because of its induction of HO-1.

### CO protects against A*β* toxicity: lack of involvement of a K^+^ channel ‘surge'

**Figure 5:**
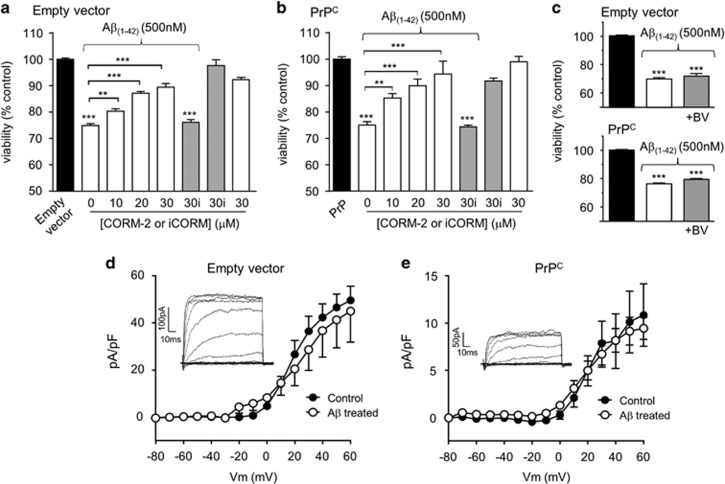
CO protects against A*β*-induced toxicity independently of K^+^ channel activity. Change in cell viability in empty vector containing (**a**) and PrP^c^-expressing (**b**) cells following a 24 h treatment with 500 nM A*β* in the absence or presence of various concentrations of the CO-donor CORM-2 (white bars). Also shown is the lack of effect of iCORM (gray bars) at 30 *μ*M. CORM or iCORM when applied alone had no effect on cell viability. Bars represent mean±S.E.M. of six repeats for the empty vector containing cells and 14 repeats for PrP^c^-expressing cells. Statistical significance is indicated by ***P<0.001, ^#^*P*<0.01 when compared to the corresponding control and corresponding A*β*_1-42_ treated cells, respectively. (**c**) Change in cell viability in empty vector containing (upper) and PrPc-expressing (lower) cells following a 24 h treatment with 500 nM A*β* in the absence or presence of 10 mM biliverdin (BV), as indicated. Bars represent the mean±S.E.M. of 4 repeats. (**d**, **e**) Mean (± S.E.M., *n*=7–9 cells in each case) current density *versus* voltage relationships determined in empty vector containing (**d**) and PrP^c^-expressing cells (**e**) with (open symbols) or without (solid symbols) exposure to 1 *μ*M A*β* for 24 h. Inset shows example families of currents, evoked by step depolarizations (to between −20  and +60 mV) applied from a holding potential of −70 mV, from which the mean plots were calculated

In order to examine whether the protective effects of HO-1 induction / over-expression could be attributable to its ability to generate CO (as suggested by the data presented in Figure 2), we examined the ability of the CO-donor, CORM-2, to protect cells against A*β* toxicity. In both empty vector containing cells (Figure 5a) and PrP^c^-expressing cells (Figure 5b), exposure of cells to CORM-2 caused a concentration-dependent reduction in the toxic effects of A*β*. The control compound, iCORM, was without significant effect, and neither CORM-2 nor iCORM affected viability when applied alone. These data support the hypothesis that HO-1 affords protection against A*β* toxicity *via* the production (and subsequent action) of CO. In further support of this hypothesis, we examined the effects of biliverdin (also produced by HO-1) on A*β* toxicity. As shown in Figure 5c, exposure of cells to 10 *μ*M biliverdin was without effect on the toxicity of A*β* in both cell groups, further supporting the proposal that CO mediates the protective effects of HO-1 in these cells.

Since exposure of cells to A*β* triggers apoptosis, we investigated whether this was also associated with increased K^+^ channel activity at the plasma membrane. Previous studies have shown this is a key event in oxidant-induced apoptotic cell death,^22, 44^ and we have shown previously that CO is protective against apoptosis triggered by a K^+^ channel ‘surge' (i.e. rapid insertion of channels into the membrane) by inhibiting the activity of these channels (specifically, Kv2.1^23, 37^). Whole-cell patch-clamp recordings revealed that K^+^ current densities, indicative of K^+^ channel activity at the plasma membrane, were not significantly affected by exposure to A*β* for 24 h in either the vector only containing cells (Figure 5d) or in the PrPc-expressing cells (Figure 5e). However, it was noted that K^+^ current density was significantly reduced in PrPc-expressing cells as compared with empty vector containing cells. The reasons for this are currently unknown. This notwithstanding, cell death induced by A*β* was not attributable to the K^+^ channel ‘surge' associated with oxidant-induced apoptosis, indicating that HO-1/CO provided protection against A*β* toxicity *via* an alternative mechanism.

### Involvement of the CaMKKII/AMPK pathway in A*β* neurotoxicity

**Figure 6:**
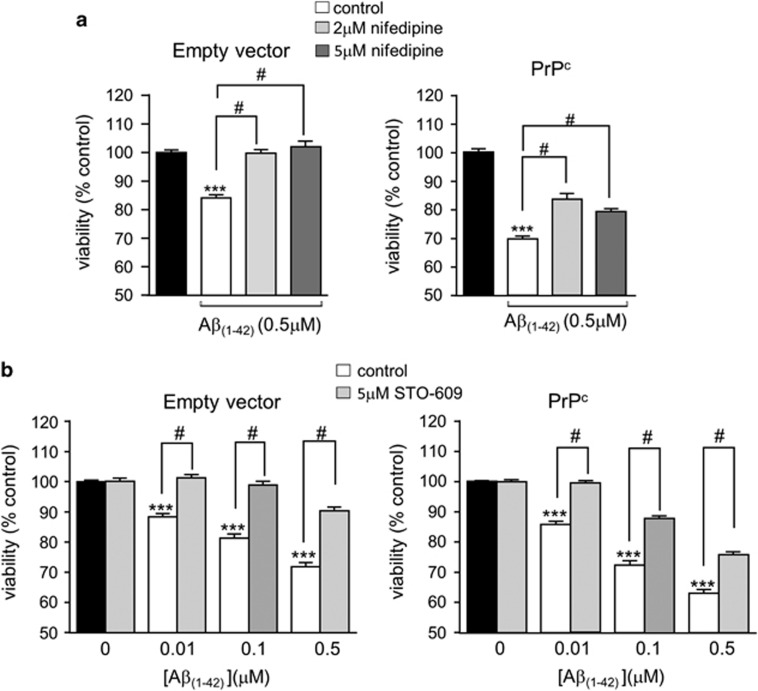
A*β* toxicity involves Ca^2+^ influx and CamKKII activation. (**a**) Cell viability monitored in empty vector containing (left) and PrPc-expressing cells (right) following exposure to A*β* applied for 24 h either alone or in the additional presence of nifedipine, as indicated. Bar graphs show the mean±S.E.M. of five repeats each for empty vector containing and PrPc-expressing cells. Statistical significance: ***P<0.001 and ^#^*P*<0.01 compared to corresponding control and corresponding A*β*_1-42_ treated cells, respectively. (**b**) Cell viability monitored in empty vector containing (left) and PrPc-expressing cells (right) following exposure to A*β* applied for 24 h either alone or in the additional presence of the CAMKK inhibitor STO-609. Black bars represent the respective untreated cells, clear bars represent cells treated with A*β* and gray bars represent cells treated with A*β* together with 5 *μ*M STO-609. Bars represent the mean±S.E.M. of four repeats for the empty vector containing and six repeats for the PrPc-expressing cells. Statistical significance is denoted by ***P<0.001, ^#^*P*<0.01 when compared with corresponding control and corresponding A*β*_1-42_ treated cells, respectively

**Figure 7:**
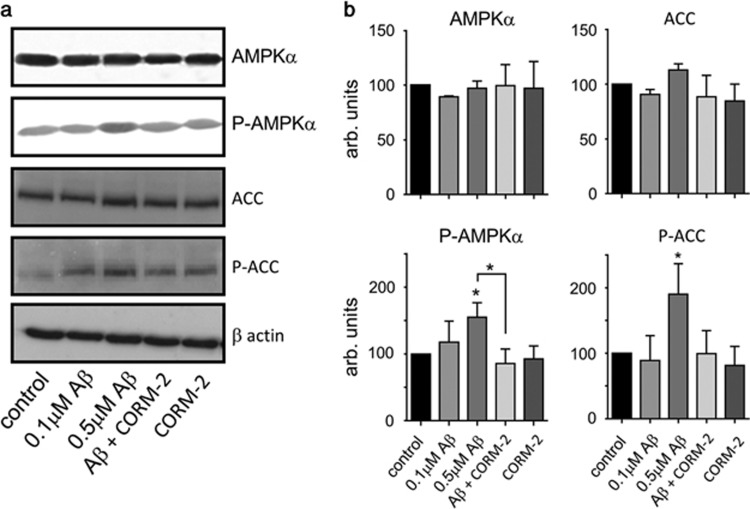
CO prevents AMPK activation by A*β*. (**a**) Example western blots showing total and phosphorylated AMPK*α*, total and phosphorylated acetyl–CoA carboxylase (ACC) and the corresponding *β* actin levels in lysates from PrP^c^-expressing cells. Cells were either untreated, or exposed to A*β* (100 and 500 nM, 24 h), or to 500 nM A*β* in the additional presence of 20 *μ*M CORM-2, or to CORM-2 alone, as indicated. (**b**) Mean (±S.E.M., from three experiments) densitometry data under each of the conditions exemplified in (**a**). Statistical significance indicated by *P<0.05 when compared with corresponding control and corresponding A*β*_1-42_ treated cells, respectively

Recent studies have suggested that A*β* neurotoxicity can involve activation of the Ca^2+^ /calmodulin kinase kinase II (CaMKKII)/AMPK pathway.^25, 26^ To investigate the involvement of this pathway in A*β*−induced neurotoxicity in SH-SY5Y cells as reported here, we first investigated the effects of suppressing Ca^2+^ influx into cells using the l-type Ca^2+^ channel inhibitor nifedipine. Nifedipine significantly attenuated A*β* toxicity in both empty vector containing cells and PrPc-expressing cells (Figure 6a), suggesting Ca^2+^ influx *via* l-type Ca^2+^ channels is involved in A*β* toxicity. Since a rise of [Ca^2+^]_i_ can lead to activation of CaMKKII, we next explored the involvement of this kinase in A*β* toxicity, and found that exposure of cells to the CaMKKII inhibitor STO-609 also significantly attenuated A*β* toxicity (Figure 6b). Exposure to both nifedipine and STO-609 produced no additional attenuation above that observed by either agent applied alone (data not shown). In agreement with recent studies, we also found that A*β* activated AMPK, as demonstrated by a specific increase in the level of AMPK phosphorylation (without change in total AMPK expression levels (Figure 7)). In further support of this, we found that A*β* increased the level of ACC phosphorylation, the major AMPK substrate. Importantly, CO (applied as CORM-2) prevented AMPK activation and subsequent phosphorylation of ACC (Figures 7a and b).

## Discussion

The present study aimed to investigate whether HO-1, *via* CO generation, provides neuroprotection against the toxicity of A*β*. There is compelling evidence to suggest that increased HO-1 expression can be neuroprotective against exposure to glutamate, oxidants or physical damage,^27, 45^ and this protection may be attributable, at least in part, to the production of CO (e.g. Zeynalov *et al.*^36^). Although much of the cellular damage associated with AD is reminiscent of oxidative damage caused by such agents/interventions, it is important to establish the specific mechanisms underlying neurodegeneration in AD, in order to understand any protective actions of HO-1/CO and so, potentially, exploit such actions in the development of new anti-neurodegenerative therapies. Indeed, whilst expression of HO-1 has long been known to be increased in AD patients,^27^ it remains to be fully resolved whether this is beneficial or detrimental. Detrimental effects arising from increased HO-1 expression specifically in glia, have been associated with excessive deposition of iron as a consequence of increased heme degradation.^46, 47, 48^ However, other studies suggest increased astrocytic HO-1 expression can provide protection for nearby neurons arising specifically from the production of CO.^49^

The present study indicates that HO-1 is indeed protective, and protection appears to arise *via* the production of CO. Our study employed SH-SY5Y cells over-expressing PrP^c^ and demonstrated that such cells were more vulnerable to the toxicity of A*β* when compared with the empty vector containing cells which lack endogenous PrP^c^ expression^50^ (Figure 1). A*β* has been show to aggregate and assemble into a range of dynamic, soluble species, and defining the particular *in vivo* ‘toxic' species which correlate most significantly with AD progression is challenging. PrP^c^ has been identified as a high-affinity receptor for oligomeric A*β.*^38, 39, 51, 52, 53^ PrP^c^ is thought to be a critical mediator of the synaptic loss, neurotoxicity, long-term potentiation impairments and memory deficits that are caused by these A*β* oligomers.^38, 51, 52^ However, the heterogeneity and lack of characterization of the synthetic A*β* preparations used in some of these studies makes it difficult to define the active PrP^c^-dependent A*β* toxic assemblies. A recent report^40^ identified protofibrils to be a key PrP^c^-specific binding species, defining their triple helical structure as A*β* nanotubes. These particular structures of A*β* caused PrP^c^-dependent synaptotoxicity.^40^ Electron microscopy indicated that our preparation of A*β* resembles those used in that particular study, and thus provide an explanation as to why our PrPc-expressing cells were selectively more vulnerable to A*β* toxicity, when their sensitivity to apoptosis induced by oxidants was similar to that seen in cells lacking PrP^c^ (Figure 1).

Several lines of evidence suggest that HO-1 provided neuroprotection, reducing the toxic effects of A*β*: thus, chemical induction of HO-1 with protoporphyrins (Figures 3a and b) or over-expression of HO-1 (Figure 3c) reduced the toxic actions of A*β*. Furthermore, protection was provided by hypoxic induction of HO-1 (Figure 4). Such hypoxic treatment can alter the expression of many proteins which might contribute to neuroprotection (see e.g.^54^ yet this effect of hypoxia was prevented by the selective HO-1 inhibitor, QC-15.^43^ Collectively, these data strongly suggest that HO-1 provides protection against the toxic actions of A*β*. Furthermore, our results also indicate that HO-1 is likely to be protective because of its ability to generate CO. Thus, although HO-1 activity was not assessed directly (e.g. *via* measurement of CO or bilirubin production) the established CO-donor, CORM-2 (but not the inactive form, iCORM) mimicked the effects of HO-1 induction (Figures 2 and 5) to provide protection against A*β* toxicity. Furthermore, another HO-1 product, biliverdin, was without protective effect (Figure 5).

Our previous work has indicated that HO-1, through the production of CO, protects hippocampal neurons from oxidant-induced apoptosis by inhibiting the voltage-gated K^+^ channel Kv2.1.^23^ Similarly, HEK293 cells over-expressing Kv2.1 displayed increased vulnerability to oxidant-induced apoptosis, and this was also prevented by CO inhibition of Kv2.1.^23^ Since earlier reports had suggested that toxic effects of A*β* might arise due to its ability to increase outward K^+^ currents,^55, 56^ we explored this as a possible mechanism by which CO might protect SH-SY5Y cells against A*β* toxicity. Perhaps surprisingly, K^+^ currents in SH-SY5Y cells were unaffected by the same levels of A*β* which induced toxicity (Figure 5). The discrepancy between this finding and earlier reports on the ability of A*β* to augment K^+^ currents is not clear at present, but it is noteworthy that previous studies employed A*β* at high (>10 *μ*M) concentrations,^55, 56^ whereas the present study largely employed sub-micromolar concentrations of A*β*. Our results indicate that K^+^ channel modulation is unlikely to account for the toxic actions of A*β* and, therefore, the protective effects of CO. Our data also contrast with an earlier report that expression of PrP^c^ is associated with augmentation of K^+^ currents carried by Kv4.2,^57^ although we do not know the contribution of Kv4.2 to whole-cell K^+^ currents in SH-SY5Y cells employed here.

In order to explore alternative pathways by which CO might afford neuroprotection, we first examined the effects of the l-type Ca^2+^ channel blocker, nifedipine. Clinical studies have shown that this class of channel blocker is beneficial in slowing down the progression of AD,^58^ and we found nifedipine to provide significant relief from A*β* toxicity (Figure 6a). This finding prompted us to investigate potential Ca^2+^-dependent pathways associated with A*β* toxicity. Several studies have implicated AMPK - which can be activated by the Ca^2+^-dependent upstream kinase, CaMKK*β* - as exerting important influences on the development of AD. Thus, for example, Thornton *et al.*,^25^ demonstrated that exposure of cortical neurones to A*β* (20 *μ*M, 30 min) activates AMPK in a CaMKK*β*-dependent manner and, importantly, that AMPK subsequently acts as a Tau kinase, contributing to its hyperphosphorylation. Yoon *et al.*^26^ also reported that A*β* activates AMPK, and indicated that this led to endoplasmic reticulum stress arising from inhibition of the mTOR pathway. This in turn led to activation of JNK3 which, crucially, phosphorylated APP (amyloid precursor protein). This phosphorylation of APP promotes its internalization and cleavage to generate increased levels of A*β*. Most recently, Ca^2+^ and CaMKK*β*-dependent AMPK activation by A*β* was confirmed in a study which also demonstrated that activation of the kinase led to a loss of dendritic spines (an early feature of AD) and also to Tau phosphorylation.^59^ Our data indicate that A*β* activates AMPK in SH-SY5Y cells (Figure 7). Furthermore, this activation is prevented by CO. The exact site at which CO acts to prevent AMPK activation, and therefore provide neuroprotection, remains to be determined. However, this is worthy of further exploration: despite its deserved reputation as a potent toxin, the physiological actions of endogenous CO, and its potential as a therapeutic agent in numerous disorders, is increasingly being recognized (e.g. Motterlini *et al.*^60^). Future studies will reveal whether it may also be of benefit in combatting neurodegenerative diseases.

## Materials and Methods

### Tissue culture

All experiments were conducted using SH-SY5Y cells stably transfected with either empty pIRES*neo* vector (BD Biosciences, Oxford, UK; termed empty vector containing cells in this study) or engineered to express murine PrP^c^ containing the 3F4 epitope tag (human M108/M111; termed PrP^c^ cells), as described previously.^50^ Cells were cultured in DMEM medium containing glutamine, supplemented with 10% (v/v) fetal calf serum, penicillin (100 U/ml) and streptomycin (100 U/ml) (all from GIBCO Life Sciences, Paisley, Scotland, UK). Cells were incubated at 37 °C in a humidified incubator gassed with 95% air and 5% CO_2_, passaged every 7 days and used up to 10 passages.

Human HO-1 over-expressing SH-SY5Y cells were generated in-house and cultured using DMEM media with glutamine, supplemented with 10% (v/v) fetal calf serum, penicillin (100 U/ml), streptomycin (100 U/ml) (all from GIBCO) and hygromycin B (200 *μ*g/ml; Calbiochem, Watford, UK). Briefly, SH-SY5Y cells were transfected with the appropriate pcDNA3.1/human HO-1 construct (Genbank Accession No.: NM_002133.2) using electroporation (Amaxa - Lonza, Slough, UK) according to manufacturer's instructions. Stably transfected cell lines were selected with hygromycin B antibiotic (200 *μ*g/ml, Calbiochem) added 3 days after transfection. Selection was applied for 4 weeks (media changed every 4-5 days), colonies were then picked, grown to confluence and screened by western blotting for HO-1 expression after culturing in T25 flasks for 24–48 h. Hygromycin B selection was maintained throughout the cloning process at 200 *μ*g/ml and in all subsequent passages once stable clones had been positively identified.

### Primary cultures of hippocampal neurones

Hippocampi of 6–8-day-old Wistar rats were removed as described previously.^39, 61^ Brain tissue was incubated with 0.25 *μ*g/ml trypsin for 15 min at 37 °C in phosphate buffered saline (PBS). Trypsin digestion was terminated by the addition of equal amounts of PBS, supplemented with 16 *μ*g/ml soybean trypsin inhibitor (type I-S; Sigma, Paisley, UK), 0.5 g/ml DNase I (type II from bovine pancreas; 125 kilounits/ml; Sigma), and 1.5 mM MgSO_4_. The tissue was then pelleted by centrifugation at 3000 × *g* for 5 min, resuspended in 2 ml of PBS with 100 *μ*g/ml soybean trypsin inhibitor, 0.5 *μ*g/ml DNase I and 1.5 mM MgSO_4_, triturated gently, and then centrifuged at 3000 × *g* for 5 min to pellet the hippocampal neurons. The pellet was resuspended in 5 ml of minimal Earle's medium supplemented with 10% (v/v) FBS, 13 mM glucose, 50 IU/ml penicillin, and 50 g/ml streptomycin and added to poly-l-lysine (1.5 mg/ml)-coated coverslips (0.5 ml/well for 24-well plates). After 24 h, the medium was topped up to 1 ml. After a further 24 h, the culture medium was replaced with one containing 10% (v/v) heat-inactivated horse serum and 80 *μ*M fluorodeoxyuridine to prevent proliferation of non-neuronal cells. After a further 24 h, the medium was replaced with serum-free Neurobasal medium, supplemented with 2% (v/v) B27, 50 IU/ml penicillin, 50 *μ*g/ml streptomycin, 80 *μ*M fluorodeoxyuridine, 25 mM glutamic acid, and 0.5 mM glutamine.

The cell media used to treat the 7 day cells consisted of neurobasal media supplemented with penicillin (100 U/ml), streptomycin (100 U/ml), 1% N-2 supplement (to prevent astrocyte growth) and 0.1% Glutamax (all from GIBCO). The media used for treating the 14 and 21 day cells was free of glutamax as it becomes toxic to the cells at this stage.

### A*β* preparation and assessment

A*β*_1-42_ and A*β*_42-1_ (r-Peptides, Bogart, GA, USA) were dissolved in DMEM (Gibco) to make up 100 *μ*M stock solutions and kept at −20 ºC. When needed for experiments one aliquot was maintained at 37 °C for 24 h to form oligomers prior to treating the cells. EM images were analyzed using FIJI (ImageJ-2). Briefly, all images were magnified (400%) using Photoshop CS5.1 (Adobe Systems Inc., Maidenhead, UK) and the longest dimension of all globular assemblies and protofibrillar structures in each resulting image were measured after setting the pixel/scale ratio.

### Cell viability assays

Cell viability was investigated using thiazolyl blue tetrazolium bromide (MTT) assays. Cells were cultured in poly-lysine coated 96-well plates to >50% confluence. The final volume of each well after any treatment was kept at 100 *μ*l. Cells were treated for 24 h with different concentrations of either A*β*_1-42_ or A*β*_42-1_ dissolved in serum-free culture media. Likewise, the media in control wells was also replaced with serum-free media for 24 h to ensure that any effects observed were due to A*β* application rather than serum withdrawal. This was done for all the experiments involving A*β* application. When applying CORM-2 for 24 h, cells were treated twice per day (9.30am and 5pm). Following 24 h treatments with A*β* and CORM-2, the media was discarded and the cells gently washed (2x) with PBS to remove all traces of CORM-2, and so avoid a direct reaction with MTT. PBS was then replaced with 100 *μ*l of fresh cell culture media in each well. 11 *μ*l of MTT solution (5 mg/ml in sterile PBS, Sigma) was then added to each well (10% by volume) and the cells incubated at 37 °C for 3 h. Post-incubation, an equal volume (111 *μ*l per well) of solubilizing solution, consisting of isopropanol and HCl (24 ml propan-1-ol/isopropyl alcohol (Sigma)+1 ml 1 M HCl), was added to each well to lyse the cells. The contents of each well were thoroughly mixed by pipetting. Absorbance was measured at 570 nm and at 630 nm using a spectrophotometer. The experiments were performed in duplicate and repeated using cells from at least 3 different passages to ensure reliability of the results. All results were normalized to untreated control cells and shown as a percentage change in cell viability compared to the corresponding controls.

To determine the proportion of cell death attributable to apoptosis, we employed the CellEvent staining protocol for both SH-SY5Y cells and hippocampal neurons. For this, cells were cultured on poly-lysine coated glass coverslips in 6-well plates as above, and then (after cells reached ca. 50% confluence) treated for 24 h with 500 nM or 1 *μ*M A*β*_1-42_, A*β*_1-42_+CORM-2, or staurosporine (applied at 1 *μ*M for 10 min). Following treatment, cell media was discarded and the CellEvent dye (8 *μ*M in PBS, Invitrogen, Paisley, UK) applied for 30 min in the dark at 37 ºC. Thereafter, following a rapid wash, cells were incubated with propidium iodide (500 nM, Invitrogen) for 5 min. Coverlips were washed again with PBS prior to applying 2 drops of Hoechst 33342 solution (Invitrogen) to 1 ml of PBS in each well, and then incubated for 20 min in the dark in order to stain nuclei. After further washing (3 × 5min in the dark), cells were fixed with 4% paraformaldehyde for 20 min, then washed gently with PBS and the coverslips mounted on slides using mounting medium (Vectashield^R^, Vector Laboratories, Peterborough, UK). The coverslips were sealed and examined using a Nikon E600 light microscope (Nikon, Kingston upon Thames, UK). All the images were obtained using the x40 lens and Q imaging micropublisher ACQuis (Syncroscopy, Cambridge, UK) software. At least three fields of interest were taken for each slide, and the number of cells with Hoechst positive nuclei that also stained positive for CellEvent or PI were counted. The data is presented as a percentage of (+) CellEvent or PI stained cells compared to the corresponding Hoechst (+) cells.

Hippocampal neurones were cultured on poly-lysine coated coverslips for 7, 14 or 21 days. On the specific days, the coverslips were washed twice with sterile PBS twice to get rid of cell debris and the cells were then treated for 24 h with fresh media for the control cells or media containing 100 nM A*β*_1-42_, 10 *μ*M CORM or 100 nM A*β*_1-42_+10 *μ*M CORM. Following the 24 h incubation period, the cells were stained with the CellEvent dye and examined and analyzed exactly as for SH-SY5Y cells.

### Immunocytochemistry

Cells were cultured on poly-lysine coated glass coverslips in 6-well plates at>50% confluence prior to treatment with either 3 *μ*M cobalt protoporphyrin (CoPPIX) or 30 *μ*M chloroferriprotoporphyrin (hemin; Calbiochem) for 24 h, or prior to exposure to hypoxia (0.5% O_2_, 48 h). Following said treatments, cells were immunostained for HO-1. Briefly, media was discarded and the cells were washed (3 × 5min) with Dulbecco's PBS. Cells were then fixed with parafomaldehyde (4% in PBS) for 20 min, following which they were permeabilized with PBS containing 0.22% Triton X100 supplemented with 10% normal goat serum (NGS; Sigma). Following 3 × 5 min washes with Dulbecco's PBS containing 1% NGS, cells were then incubated overnight at 4 °C with the primary antibody; rabbit polyclonal anti-HO-1 (1 : 100, Santa Cruz, Heidelburg, Germany) in Dulbecco's PBS containing 1% NGS. The following day, cells were washed with Dulbecco's PBS containing 1% NGS (3 × 5 min). Antibody binding was visualized by incubating the cells with a secondary antibody; Alexa Fluor-488 conjugated anti-rabbit IgG (1 : 1000, Invitrogen), for 1h in the dark. Post-incubation, and following 3 × 5 min washes with Dulbecco's PBS, coverslips were mounted on slides using Vectashield^R^ mounting media containing DAPI (Vector Laboratories). The slides were then examined using a Zeiss (Cambridge, UK) laser scanning confocal microscope (LSM 510).

### Electrophysiology

Fragments of coverslip with attached cells were transferred to a continuously perfused (3–5 ml/min) recording chamber mounted on the stage of an Olympus (Southend, UK) CK40 inverted microscope. All experiments were carried out at 22±1 °C, unless otherwise stated. Cells were continually perfused with a solution containing (in mM): 135 NaCl, 5 KCl, 1.2 MgCl_2_, 5 HEPES, 2.5 CaCl_2_, 10 D-glucose (pH 7.4 with NaOH). Whole-cell patch-clamp recordings were then obtained in voltage-clamp mode with cells clamped at −70 mV. Patch pipettes had resistances 4-6 MΩ when filled with an intracellular solution consisting of (in mM): 10 NaCl, 117 KCl, 2 MgCl_2_, 11 HEPES, 11 EGTA, 1 CaCl_2_, 2 Na_2_ATP (pH 7.2 with KOH). After breaking into the whole-cell configuration, series resistance was monitored throughout the duration of experiments. If a significant increase occurred (>20%), the experiment was terminated. Signals were acquired using a Axopatch 200B (Axon Instruments Inc., Foster City, CA, USA) controlled by Clampex 9.0 software *via* a Digidata 1322A interface (Axon Instruments Inc.). Data were filtered at 1 kHz and digitized at 2 kHz. To evoke ionic outward K^+^ currents in SHSY5Y cells, a series of 100 ms depolarizing steps from −80  to +60 mV, in 10 mV increments, were employed. Offline analysis was carried out using the data analysis package Clampfit 9 (Axon Instruments) and data are expressed as mean±S.E.M.

### Western blotting

Cells used for immunoblotting were cultured in T25 flasks and when confluent, washed in PBS and then lysed *in situ* with 200 *μ*l of mammalian protein extraction reagent (M-PER, Pierce, Loughborough, UK) containing complete protease inhibitor tablets (Roche) for 30 min at room temperature. Protein levels in the lysates were assessed using a BCA assay (Pierce). Cell proteins (typically 30 *μ*g protein per lane) were separated on 12.5%, 0.75 mm thick polyacrylamide SDS gels and electrophoretically transferred to 0.2 *μ*m PVDF membranes (BioRad). The blots were probed with primary antibodies (1 : 1000, cell signaling) raised against AMPK*α*, phospho-AMPK*α*, Acetyl-CoA Carboxylase (ACC), Phospho-acetyl-CoA Carboxylase (phospho-ACC) at room temperature for 3 h or HO-1 (1 : 200, Santa Cruz) at 4 °C overnight. Next, membranes were washed with PBS for 30 min prior to incubating with the appropriate anti-rabbit or anti-mouse horse radish peroxidise-conjugated secondary antibody (1 : 2000; Amersham Pharmacia Biotech, Buckinghamshire, UK) for 1 h at room temperature. Following this incubation, membranes were washed in PBS for 30 min and bands visualized using an enhanced chemiluminescence detection system and hyperfilm ECL (Merck, UK).

### Statistical analysis

Data are shown as mean±S.E.M. Statistical analysis was carried out using one-way ANOVA followed by either the Dunnett's or Bonferroni post-test, as appropriate. *P* values of less than 0.05 were considered significant. CellEvent results were analyzed using a two-way ANOVA followed by a Bonferroni post-test. *P*<0.05 was considered to be significant.

## Abbreviations

A*β*: amyloid *β* peptide (A*β*)
AD: Alzheimer's disease (AD),
AMPK: AMP-dependent protein kinase (AMPK)
CaMKKII: Ca^2+^/calmodulin kinase kinase II ()
CoPPIX: cobalt protoporphyrin
CORM-2: carbon monoxide releasing molecule
DMEM: Dulbecco's minimum essential medium
DTDP: dithiodipyridine (DTDP)
HO-1: Heme oxygenase-1 (HO-1)
MTT: thiazolyl blue tetrazolium bromide
PI: propidium iodide
PBS: phosphate buffered saline
